# Maternal And preGnancy hEalth duriNg elevaTed heAt – MAGENTA: Novel data linkages and methods to understand the impacts of climate change on deprived communities in Wales and London.

**DOI:** 10.23889/ijpds.v9i5.2572

**Published:** 2024-09-18

**Authors:** Vincent Nguyen, Kate Lewis, Lorraine Dearden, Bianca De Stavola

**Affiliations:** 1 University College London

## Introduction

Without accounting for the underlying need for Special Educational Needs (SEN) provision, pupils with SEN in England have been shown to have higher rates of unauthorised absences.

## Approach

Our objective was to understand the impact of SEN provision on unauthorised absences in children with an expected need for SEN, those born with Cleft Lip and/or Palate. We used linked health and educational records to investigate the relationship between three categories of SEN provision (No Provision versus SEN Support versus Education and Healthcare Plan (EHCP)) and unauthorised absences. We aimed to causally estimate the incident rate ratio (IRR) of SEN Support vs No Provision and EHCP vs SEN support on unauthorised absences between years one and six of primary education. Combining sociodemographic, educational and clinical variables from linked data, we used propensity-score based methods to adjust for non-randomised assignment to these categories of treatment.

## Conclusions

Between academic years 2009 to 2019, we found common support between comparison groups No provision (n = 4350) and SEN support (n = 2009) only for children with Cleft lip and palate and only reported on these comparisons. Unadjusted estimates demonstrate a 22% increase in absences in children with SEN support over No Support (IRR: 1.22 95%CI: 1.10-1.34). Reweighting for need using data from linked health and education records demonstrated a 14% reduction in unauthorised absences (IRR: 0.86 95%CI: 0.76-0.97).

## Implication

Contrary to prior findings, by carefully matching on detailed linked health and education characteristics, we find SEN provision reduces unauthorised absences.

